# ERCP: Energy-Efficient and Reliable-Aware Clustering Protocol for Wireless Sensor Networks

**DOI:** 10.3390/s22228950

**Published:** 2022-11-18

**Authors:** Fatma H. El-Fouly, Ahmed Y. Khedr, Md. Haidar Sharif, Eissa Jaber Alreshidi, Kusum Yadav, Huseyin Kusetogullari, Rabie A. Ramadan

**Affiliations:** 1 Department of Communication and Computer Engineering, Higher Institute of Engineering, El-Shorouk Academy, El-Shorouk City 11837, Egypt; 2 College of Computer Science and Engineering, University of Ha’il, Ha’il 8650, Saudi Arabia; 3 Systems and Computer Engineering Department, Faculty of Engineering, Al-Azhar University, Cairo 11651, Egypt; 4 Department of ICT, University of Agder, 4630 Kristiansand, Norway; 5 Department of Computer Science, Blekinge Institute of Technology, 37141 Karlskrona, Sweden; 6 Computer Engineering Department, Faculty of Engineering, Cairo University, Giza 12613, Egypt

**Keywords:** wireless sensor networks, clustering, routing, reliability, energy balance

## Abstract

Wireless Sensor Networks (WSNs) have been around for over a decade and have been used in many important applications. Energy and reliability are two of the major problems with these kinds of applications. Reliable data delivery is an important issue in WSNs because it is a key part of how well data are sent. At the same time, energy consumption in battery-based sensors is another challenge. Therefore, efficient clustering and routing are techniques that can be used to save sensors energy and guarantee reliable message delivery. With this in mind, this paper develops an energy-efficient and reliable clustering protocol (ERCP) for WSNs. First, an efficient clustering technique is proposed for sensor nodes’ energy savings considering different clustering parameters, including the link quality metric, the energy, the distance to neighbors, the distance to the sink node, and the cluster load metric. The proposed routing protocol works based on the concept of a reliable inter-cluster routing technique that saves energy. The routing decisions are made based on different parameters, such as the energy balance metric, the distance to the sink node, and the wireless link quality. Many experiments and analyses are examined to determine how well the ERCP performs. The experiment results showed that the ECRP protocol performs much better than some of the recent algorithms in both homogeneous and heterogeneous networks.

## 1. Introduction

A Wireless Sensor Network (WSN) refers to an infrastructure-less system of numerous tiny devices called sensor nodes. Such nodes are responsible for monitoring the area of interest and communicating with each other via wireless links to send their collected information to the sink node. However, the WSN is known to be a highly resource-constrained network, in which the energy constraint is always the main issue that affects the network operation. This results in researchers’ primary concerns in this field being energy conservation and the network’s lifetime [1,2,3].

Indeed, communication is the major source of energy consumption in WSNs. For this reason, numerous research publications have examined the energy-aware routing problem in WSNs [4,5]. Among massive energy-aware routing techniques, cluster-based routing is one of the most effective solutions that can conserve sensor nodes’ energy and thus increase the network’s lifetime. In the clustering process, the network is divided into groups. Each group has a head or leader. Such cluster heads collect sensor nodes’ local data, aggregate it, and transfer it to the sink node directly or via other cluster heads [6]. However, the cluster heads may lose their energy faster than the conventional sensor nodes due to the extra load of receiving, aggregating, and transferring data to the sink node. Hence, the cluster heads should be energy-efficient nodes because of their transmission and reception responsibilities. The death of such nodes in an area would cause network partitioning, and, thus, important data may be lost. Therefore, the proper selection of cluster heads plays a vital role in conserving sensor nodes’ energy and enhancing the network’s lifetime. In this context, in many existing clustering techniques, the cluster head assignment depends on the average cluster distance to maximize cluster heads’ lifetimes. Therefore, the cluster heads are placed near their member nodes. Certainly, if a cluster head dies, the network may suffer from severe energy unbalance. Consequently, designing an energy-balanced clustering strategy is highly needed to optimize the network’s lifetime [7,8].

In view of the above advantages of the clustering techniques, numerous cluster head election strategies have been suggested in the literature for energy consumption conservation and network lifetime enhancement. Most of them tried optimizing energy efficiency by employing nodes’ energy in the cluster head decision to address the energy unbalance problem [3,9,10,11,12,13,14,15,16,17,18,19,20,21,22,23,24]. However, from our point of view, in addition to energy, reliability is another factor that needs careful consideration when designing any clustering algorithm. The quality of wireless links enables sensor nodes to communicate with each other; thus, reliable wireless links would be required to successfully deliver data packets from the member nodes to their cluster heads. However, due to wireless communication’s inherent nature in WSNs, packet losses are quite inevitable. Wireless connections are prone to network disturbances due to several environmental parameters such as interference and fading. This increases the retransmission possibility of lost packets and thus causes more energy consumption and more delivery delay. This eventually affects the network’s throughput and lifetime [25,26,27,28].

To address the problems mentioned above, the work in this paper involves two phases. In the first phase, a cluster head is chosen, while the second phase proposes an inter-cluster routing algorithm. The proposed framework considers energy efficiency and network reliability to optimize the network’s lifetime and throughput. The major contributions are as follows:

(1) Propose a novel energy-efficient and reliable clustering algorithm considering nodes’ residual energy, quality of wireless links, cluster head load, and distance representing the average intra-cluster distance and distance to sink node.
(2) Propose an energy-efficient reliable-aware routing algorithm considering the link quality, distance to sink node, nodes’ residual energy, and load balancing.

The paper outline is as follows: The relevant work to this topic is included in Section 2. The modeling of the problem is described in Section 3. The details of the proposed algorithm are presented in Section 4, whereas the results are covered in Section 5. The paper’s conclusions are highlighted in Section 6.

## 2. Related Work

This section discusses different clustering strategies developed for maximizing the WSN’s lifetime. To our knowledge, most of them are mainly focused on an energy-efficient scheme in which the optimal set of cluster head nodes is determined by employing the nodes’ energy to achieve a balance in energy consumption and optimize the network’s lifetime [3,9,10,11,12,13,14,15,16,17,18,19,20,21,22,23,24]. However, from a reliability viewpoint, such algorithms cannot have any reaction to the dynamic nature of wireless communication links. Therefore, in this section, we first explain some relevant studies that were developed for energy efficiency and network lifetime optimization [3,9,10,11,12,13,14,15,16,17,18,19,20,21,22,23,24]. Finally, we present a discussion of the differences from our proposal.

LEACH is the classical clustering technique for WSNs [18]. In every round, cluster head nodes are randomly selected to optimize the network’s lifetime. Hence, all nodes have the same opportunity to be the cluster head. Meanwhile, as the assignment of cluster head nodes is performed randomly, it is possible to choose the nodes with low energy as cluster head nodes, resulting in an unbalance in energy consumption which ultimately degrades the network’s lifetime. Therefore, several types of research have been developed over time that aim to enhance LEACH performance [19,20,21,22,23]. In [19,20,21,22,23], the authors improve the basic LEACH protocol by considering the energy factor in deciding cluster head nodes.

FR-LEACH is presented in [3]. It developed an adaptive fuzzy-based technique in which the decision to select cluster head nodes was made by modifying the basic LEACH threshold by considering both nodes’ residual energy and optimum cluster number. Through the initial phase, the energy is at its maximum level; thus, there is a greater cluster number during this period. However, energy has to be depleted with time, and the cluster number also decreases. That is to say, using the fuzzy rule, this algorithm tracks the variation in the network’s energy in each round, which helps to maintain a balance in energy consumption that optimizes the network’s lifetime.

Distance-aware residual energy-efficient SEP for WSN (DARE-SEP) is presented in [9]. The DARE-SEP locates the cluster head nodes by combining the energy of the sensor nodes and the Euclidean distance to the sink node. The energy factor balances the energy consumption of sensor nodes, maximizing network longevity. Finally, the distance factor assists in the preservation of data transmission energy to the sink node.

The hybridization of the meta-heuristic method for the dynamic cluster-based routing protocol (HMBCR) in WSNs is reported in [10]. In HMBCR, BSO-LD clustering assigns cluster head nodes, and WWO-HC routing determines optimum routing routes. The distance to neighbors, energy, distance to sink nodes, and network load determine cluster head nodes in BSO-LD. Cluster heads are selected based on the remaining node energy to balance sensor node energies. Distance factors reduce the routing path communication distance to conserve energy and increase the network life. The network load manages the cluster head load. After selecting clusters, WWO-HC assigns the appropriate data transfer path to the sink node. The WWO-HC produces a fitness function with two parameters, energy and distance. The energy parameter helps select high-energy nodes as the next hop, while the distance parameter minimizes energy use.

In [11], an energy-aware cluster-based routing protocol (ECRP) is proposed for WSNs. The energy-aware clustering and routing problem design are addressed through the ECRP protocol introduced in [11]. Initially, the ECRP adapts the same threshold of the basic LEACH in the cluster head assignment, and then the assignment is performed through a cost function based on two main factors, energy and distance, to minimize and balance energy dissipation. After the clustering, the data-forwarding phase begins. In such a phase, the assignment of the next-hop forwarding node is also performed through a cost function involving the energy and distance parameters.

In [12], a multi-hop clustering routing protocol utilizing a CRCGA is designed to increase energy efficiency and load balancing. In CRCGA, an improved chaotic genetic algorithm is employed to solve the clustering problem, aiming to save energy dissipation and distribute the load among cluster heads in a balanced way. Furthermore, the round time is determined in a new adaptive way to conserve further energy in the network.

Ref. [13] introduces a clustering-based mobile routing algorithm for WSNs. A mobile sink divides the network into segments, and the sensor nodes in each segment create a cluster. The mobile sink allocates each cluster’s head based on nodes’ energy and distance from the cluster center by utilizing greedy and ANN approaches. That is to say, the node with the highest energy and the nearest to the cluster center is assigned as a cluster head. After the clusters are generated, the mobile sink travels around each cluster center on a predetermined route to start the data-transfer process. A genetic algorithm (GA) determines such a route, so the mobile sink travels to all clusters via the shortest route.

A meta-heuristic clustering technique (CPMA) is given in [14] to maximize the network’s lifetime. The CPMA method used the Harmony Search (HS) algorithm for cluster head selection. The HS elects cluster head nodes using a fitness function that combines consumed energy and the expected ratio of energy distribution to minimize the consumed energy and smooth network energy distribution.

ML-TSEP is developed in [15]. The modified threshold value integrated the original LEACH threshold with the sensor node energy, distance to sink node, neighbor node number, and the number of times a node operates as a cluster head to minimize energy consumption and enhance network longevity.

The Distance and Energy-Aware Stable Election Routing Protocol (DE-SEP) for a heterogeneous WSN was developed [16]. In DE-SEP, the cluster heads assignment procedure prioritizes the node with the maximum energy that is nearest to the sink node for energy saving and network lifetime improvements. Finally, the DE-SEP computes the optimal number of cluster heads to prevent redundant cluster formation and minimize the consumed energy.

Gateway Clustering Energy-Efficient Centroid (GCEEC) routing minimizes energy use and cluster head load [17]. To minimize consumed energy and maximize cluster head coverage, the GCEEC protocol selects and rotates the cluster head near the cluster’s energy centroid location. Moreover, it selects a gateway node from each cluster to forward the data towards the sink node, which minimizes cluster heads’ load. After the cluster heads and gateways are identified, the data-transfer process starts. In such a process, once the cluster heads have aggregated the data from their members, it is further sent toward the sink node via gateway nodes or directly according to a threshold distance.

Enhanced Particle Swarm Optimization-based Clustering Energy Optimization (EPSO-CEO) is a technique that was developed for wireless sensor networks [24]. The PSO is adopted to construct clusters within the sink node in a centralized way and assign cluster heads in a distributed manner. In the clustering process, the nodes’ energy and distance are considered while determining the clustering fitness function to minimize and balance consumed energy. After clustering, a multi-hop routing strategy is adopted for data transfer to the sink node. The assignment of intermediate cluster heads for data routing depends on a cost function so that the node with a minimum cost is chosen as an intermediate node. Such a function depends on energy and distance factors, where the node with minimum distance and higher energy should be chosen as the next hop.

Although prior research on similar algorithms in [3,9,10,11,12,13,14,15,16,17,18,19,20,21,22,23,24] has successfully reduced the energy consumed to optimize the network’s lifetime, they have not accounted for lossy links caused by fading and interference. Ignoring such a problem might increase data loss, retransmission delay, and energy waste.

Motivated by the above discussion, we propose an energy-efficient and reliability-aware clustering protocol (ERCP). The ERCP involves two levels, the cluster head selection and the inter-cluster routing protocol. The cluster head selection considers the average intra-cluster distance, cluster load, residual energy, and reliability of intra-cluster data transfer. To achieve reliable intra-cluster data transfer, a new link quality metric function is proposed to express the quality of wireless connections between each candidate cluster head and its member nodes. In the cluster hierarchy, aggregating the data by each cluster head from its members causes imbalanced energy loss. To address such a problem, in addition to energy balance, it is essential to balance the load among cluster heads. The cluster load is used as another factor in the cluster head selection to balance load among cluster heads. We assess the average intra-cluster distance and nodes’ energy to improve end-to-end latency and energy efficiency, as with many previous clustering approaches. The second level of this work is the inter-cluster routing protocol, which will be invoked after the clustering phase for data transfer to the sink node. It considers three main parameters: Link quality, nodes’ energy, and distance to sink node to optimize network energy efficiency and throughput. Link quality is considered to prevent data forwarding via unstable paths. This is a novel function that combines sensor node residual energy and traffic load and balances the energy usage. The distance factor is considered to minimize the consumed energy and delivery delay. Therefore, the suggested routing approach uses more realistic parameters than prior systems. Table 1 provides an overall comparison of the above-mentioned algorithms.

## 3. Problem Modelling

This section describes the research problems, and our primary goals are explained. Consider that a field *F*(*A*) is monitored by a number of nodes for a time horizon *T*. The sensor nodes were static and location-aware. The network topology is built on clusters, and sensors are organized as clusters. The cluster head is responsible for gathering data from its specific cluster using a single-hop communication channel.

Efficient exploration of the sensor network field is represented by the undirected weighted graph *G* (*V*, *E*), where *V* is the collection of nodes and *E* is the set of edges where *x*, *y* ∈ *V*. The edges represent communication links between nodes. A connection exists between nodes *x* and *y* only if they are able to communicate. Additionally, the Packet Reception Rate (*PRR*) is offered by the MAC layer [29] to measure the connection quality.

First, we start with one of the paper’s goals: Identifying an ideal cluster head that conserves nodes’ energy network lifetime extension. To achieve this, the cluster head should satisfy some constraints, including:

(1) Provides the shortest average intra-cluster distance.
(2) Provides the highest possible data transfer reliability.
(3) Provides the shortest inter-cluster distance (the distance to the sink node).
(4) Has the highest energy level.
(5) Has the minimum cluster load metric value.

Secondly, designing a routing technique aims to save energy and optimize the network’s lifetime. Furthermore, energy management is considered while designing the proposed protocol. It also examines how to avoid unreliable routing paths. To attain this aim, the proposed route must fulfill the following criteria:

(1) Minimum communication distance.
(2) Maximum reliability.
(3) In order to establish a better energy balance, the nodes participating in such a path have the highest value resulting from the new proposed energy load function.

## 4. Proposed ERCP Clustering Algorithm

This section describes the ERCP protocol, which is designed to optimize the network’s lifetime and throughput. The ERCP method works on two levels. The cluster head election and rotation constitute the initial stage of the clustering method. The second level involves the delivery of data to the sink node via an inter-cluster routing method.

### 4.1. Cluster Head Selection

This section discusses an energy-efficient and reliable clustering design. For each node *x*, its candidate neighbor set *NEB_x_* nodes not covered by cluster heads are added to the final candidate member set. This is formally represented in Equation (1),

(1) $$NM_{x}=\left\{y|y\in NEB_{x},\Gamma_{y}=1\right\}$$

where

(2) $$\Gamma_{y}=\{\begin{array}{cc} 1 & \;if\;node\;y\;is\;not\;covered\;by\;any\;cluster\;head\\ 0 & otherwise \end{array}$$

where *NM_x_* is the neighbor set of sensor node *x* that is not covered by any cluster head.

As reliable data transfer is the premise to guarantee the network’s normal operation and is the basis for optimizing WSN performance, reliable wireless links would be needed for the successful delivery of data from the member nodes to their cluster heads. However, in WSN, packet losses are rather inevitable since it is deployed in harsh environmental conditions. Retransmitting missing packets takes additional time and energy, reducing data delivery and network lifetime [24,25,26,27]. The proposed clustering technique integrates the link quality into the selection decision of the cluster heads to overcome such problems and improve the network throughput. Moreover, the retransmission reduction decreases consumed energy, which enhances the network’s lifetime. Equation (3) presents the proposed new link quality metric of candidate cluster head *x* at time *t*.

(3) $$QM_{x}\left(t\right)=\sum_{l\in NM_{x}}\left(1-PRR_{xl}\left(t\right)\right)$$

where *PRR_xl_* is the packet reception ratio for the link (*x*, *l*). According to Equation (3), the new quality metric function is designed to evaluate the quality of links between the candidate cluster head *x* and its neighbor nodes *NEB_x_*. Moreover, this is designed by the fact that the maximum link quality value is one. Hence, through the quality metric function, the closer the link quality value is to one, the lower the resulting value for the metric function, leading to choosing the cluster head that can provide more reliable links for data transfer from its members.

In the hierarchical clustering approach, every cluster assigns a cluster head that receives and aggregates the data from its members and forwards it to sink thereafter. Thus, cluster heads may lose energy faster than conventional sensor nodes. Therefore, to balance energy consumption across all nodes and extend the network’s lifetime, the clustering must avoid low-residual-energy nodes. The clustering strategy needs to avoid nodes with small residual energy. Therefore, the candidate cluster head’s energy is used as an energy metric, which is represented by Equation (4).

(4) $$EM_{x}\left(t\right)=RE_{x}\left(t\right)$$

where *RE_x_*(*t*) is the residual energy of node *x*.

To optimize delivery delay, the average intra-cluster distance is assessed in the election decision of cluster head nodes. This is defined for sensor node *x* by Equation (5) as follows:

(5) ICDx=∑y∈NMxEDxy|NMx|

where |.| denotes the size of a set and *ED_xy_* is the Euclidian distance between nodes *x* and *y*.

In addition to energy balancing, the clustering process should evenly distribute the load associated with being a cluster head among the nodes to achieve a balanced load distribution [12]. Hence, the cluster head nodes should be carefully chosen to construct well-balanced clusters. Therefore, load balancing is another factor that has to be considered in the cluster head election. In this paper, the load balancing is decided by the cluster load metric. Equation (6) gives the cluster load metric of node *y* at time interval *t* as follows:

(6) $${CL}_{x}\left(t\right)=\sum_{n=1}^{t-1}\eta_{x}^{n}$$

where

(7) $$\eta_{x}^{n}=\{\begin{array}{cc} 1 & \;if\;node\;x\;is\;chosen\;as\;a\;cluster\;head\;during\;time\;interval,\;n\\ 0 & otherwise \end{array}$$

According to Equation (6), the cluster load of node *x* at a specific time interval, *t*, is the number of times it was chosen as a cluster head from the start of network operation until *t*1.

ERCP’s clustering technique is two-staged. First, sensor nodes are deployed and switched on with the same initial energy. Sensor nodes transmit “hello” messages that contain the location, ID, and energy. Nodes construct neighbor tables after exchanging messages. The first cluster head is the node with the most neighbors. The first cluster head is selected based on heterogeneous network node residual energy.

Advanced and super nodes are given preference to become cluster heads as they have more energy. A unique cost function elects the cluster head. The new cost function is designed to identify the best cluster heads to reduce and balance energy consumption and increase network dependability. The cost function may pick the optimal node even if distance, quality, cluster load, and energy have different metrics. During each time interval *t*, the sink node broadcasts an advertisement message to trigger cluster head rotation. After scanning its neighbors, the node with the highest cost becomes the cluster’s head. Equation (8) defines the cost of selecting node *x* as the cluster head.

(8) $$CHCost_{x}(t)=\frac{EM_{x}\left(t\right)}{{ICD}_{x}*{ED}_{\left(x,sink\right)}*\left({CL}_{x}\left(t\right)+1\right)*\left(1+QM_{x}\left(t\right)\right)}$$

As given in Equation (6), the cost function is designed so that the node that has a lower quality metric will have more chance of being the cluster head, as it has a better link quality metric than other candidates. Furthermore, the node that has a higher energy metric has more chance of being the next cluster head as this node will have higher energy. Finally, the node with the minimum average intra-cluster and inter-cluster distance is more likely to be picked as a cluster head. The pseudo-code of the ERCP cluster head selection algorithm is given as Algorithm 1.
**Algorithm 1**: ERCP cluster head selection algorithm1: *ch is the* cluster head ID; 2: *NEB_x_* is the neighbor set of sensor node *x*. 3: next_cluster head [ ] is the array containing the selected cluster head nodes; 4: *NM_x_*[ ] is the array containing the neighbor nodes of sensor node x that is not covered by any cluster head; 5*: y* is the neighbor node; 6*: CH is the* number of candidate cluster head nodes; 7*: R[CH]* is the array for sorting probability amount of candidate cluster heads; **Proc 1: Candidate member nodes calculation** 8: Node *x* sends the “member” message to its all neighbors *NEB_x_*; 9: When a response is received from a node *y*, it does: 10:    if *y* is not covered by any cluster head 11:      then add *y* to *NM_x_* array 12: Endproc **Proc 2: Decision Making** 13: Node *x* sends “join” message to its neighbors with the value of its *CHCost_x_(t)* as given in *Equation (8)*; 14: $R[\;]\overset{}{\leftarrow }{\mathrm{CHCost}}_{x}(t);$ 15: $R_{\max}=0;$ 16: For (*n = 0; n = CH; n++*) 17:    If (*R[n] > R_max_*) 18:       $R_{\max}=R[n];$ 19:       $ch=x.\;R_{\max};$ 20:       next_cluster head [ ] ← *x*; 21:     EndIf 22: EndFor 21: Endproc

### 4.2. Inter-Cluster Routing Protocol

After clustering, the cluster heads have to deliver their collected data to the sink node; thus, the inter-cluster routing protocol is invoked.

Actually, communication energy is considered the major source of energy consumed in WSNs. Since it mainly depends on the communication distance, the shortest path substantially minimizes the consumed energy. However, it may result that the shortest path approach does not prolong the network’s lifetime. The network’s energy usage must be balanced for energy-efficient routing.

Relying entirely on sensor nodes’ residual energy is not the ideal way to establish network energy balance. To achieve a better energy balance, the routing protocol must refrain from using low-energy, high-traffic nodes as next hops. Using the recommended new function, the energy load function, it is feasible to include the remaining energy and traffic load of sensor nodes and let them significantly impact choosing the next hop.

The total number of messages sent by each cluster head should match that of the node. This should contain the number of messages from each cluster head member and other cluster heads to relay. That is to say, the cluster head with higher energy and less load is preferable to be chosen as a relay node. Equation (9) presents the proposed relay energy metric of cluster head *y* at time *t*.

(9) $$REM_{y}(t)=\exp\left(\frac{RE_{y}(t)-\left(NDR_{y}(t)*HE_{xy}\right)}{IE_{y}}\right)$$

where is the traffic load of cluster head *y* and *HE_xy_* is the single-hop transmission energy from *x* to *y*.

Equation (9) suggested the new energy consumption load function expresses cluster head *y’s* energy use after sending all messages. Any tiny change in the exponential function input causes a huge output change. Through an exponential function, a slight change in nodes’ energy leads to the selection of the most energy-efficient relay node [30].

In ERCP, after collecting the data from its member nodes, every cluster head looks at the cluster heads of its neighbors and unicasts the data to the best one using the cost function. The data are then routed via this neighbor, which has chosen the best cluster head among its neighbors until it reaches the sink node. Several parameters, such as the link quality, energy metric, and distance to the sink node, are used to compute the cost function. To achieve an energy consumption balance, the node with the maximum energy metric value should be treated as a relay node. Furthermore, the node with the minimum distance should be chosen as a relay node to minimize energy consumption and delivery delay. In addition, the node that achieves the best link quality should be chosen as a relay node to avoid unreliable links. The cost of cluster head *x* selecting cluster head *y* as a relay node is defined as follows:

(10) $$RCost_{xy}(t)=\left(REM_{y}(t)+\frac{1}{ED_{\left(y,sink\right)}}\right)*PRR_{xy}\left(t\right)$$

According to Equation (10), the cluster head with the maximum cost value will be selected as a relay node. The pseudo-code of the ERCP inter-cluster routing algorithm is given as Algorithm 2.
**Algorithm 2:** ERCP next hop selection algorithm1: *x* = Relay node ID; 2*: y* = Next relay node; 3: *next_hop[ ] = Array containing the selected relay nodes;* 4*: X* = The number of neighbors located in the direction of sink node; 5: *P[X]* = Array for sorting probability amount of neighbors; **Proc 1: ERCP-Next-Hop-Selection** 6: Node *x* sends “next hop selection message” to its cluster head neighbors *NEB_x_*; 7: Each node $y\in NEB_{x}$ sends reply with the current *RE_y_(t)*, *PRR_xy_(t)*, *NDR_y_(t)*; 8: For each $y\in NEB_{x}$ do 9:  If (*ED_(y,sink)_* ≥ *ED_(x,sink)_*|y∈next_hop[ ])) 10:     discard the reply message; 11:  Else 12:     calculates the cost *RCost_xy_(t)* of each *y based on Equation (9) and Equation (10)*; 13:     $P[\;]\leftarrow {RCost}_{xy}(t);$ 14:  Endif 15: EndFor 16:$P_{\max}=0;$ 17: For (*r = 0; r = X; r++*) 18:  If (*P[r] > P_max_*) 19:    $P_{\max}=P[r];$ 20:    $x=y\;.\;P_{\max};$ 21:    next_hop[ ]= *y* 22:  EndIf 23: EndFor 24: EndProc

## 5. Performance Evaluations

In this section, numerical simulation experiments are carried out to evaluate our proposal’s performance. First, the evaluation criteria are defined. Then, the evaluation methodology is described. Finally, the simulation results and comparisons with benchmarks are discussed.

### 5.1. Performance Evaluation Criteria

Four quantitative criteria are selected to evaluate the proposed approach’s performance. These evaluation criteria are explained as follows:

1. Network Lifetime [31] is the amount of time that has passed since the network started running until the first node in the network stops working because its battery is depleted.
2. The packet delivery rate (PDR) [31] is the ratio of the number of successful messages sent by the source nodes that the sink node received.
3. The average end-to-end delay [31] is the average amount of time it takes for a data packet to travel from the source node to the sink.
4. EIF, or the Energy Imbalance Factor [31], is the average difference in energy between the nodes in the whole network.
  $$EIF=\sqrt{\frac{1}{n}\sum_{i=1}^{n}{\left(RE_{i}-RE_{avg}\right)}^{2}}$$
  where *n* is the number of nodes, *RE_i_* is the node’s *i* residual energy, and *RE_avg_* is all nodes’ average residual energy.

### 5.2. Simulation Model

A series of tests are run using the MATLAB tool to evaluate our proposed techniques thoroughly. The tests are run on an Intel Core i5 dual-core CPU with a clock speed of 2.3 GHz, 4 GB of RAM, and the Windows 7 operating system. The simulation environment consists of 300 sensor nodes and a sink node in a 1000 m × 1000 m squared area. All sensor and sink nodes are expected to be deployed randomly in a field area and remain stationary after deployment. The sink node is likewise assumed to be placed at (1000, 0) m. The data flow is generated using a Poisson process with a mean parameter, λ. Furthermore, the WSN lossy connections model used in this research is described in [32]. The simulation parameters are summarized in Table 2.

We employed the energy consumption model of [32] in our experiments.

### 5.3. Simulation Results

For the verification of the feasibility and efficacy of our solution, its network lifetime, end-to-end delay, packet delivery ratio, energy imbalance factor, and time complexity are compared with the HMBCR [10], ECRP [11], and EPSO-CEO [24]. In order to better evaluate the network performance, the sensor nodes deployed in the network are considered homogeneous in some of the experiments and heterogeneous in other experiments. The heterogeneous sensors mean that sensors’ battery energies might be different from each other. In a homogeneous network, the sensor nodes have the same initial energy of 200 mJ. In a heterogeneous network, the nodes’ initial energy is random between 175 mJ and 200 mJ. In the first three experiments, the typical traffic rate ranges anywhere from 3 to 11 packets per second on average.

#### 5.3.1. Network Lifetime Evaluation

**Figure 1:**
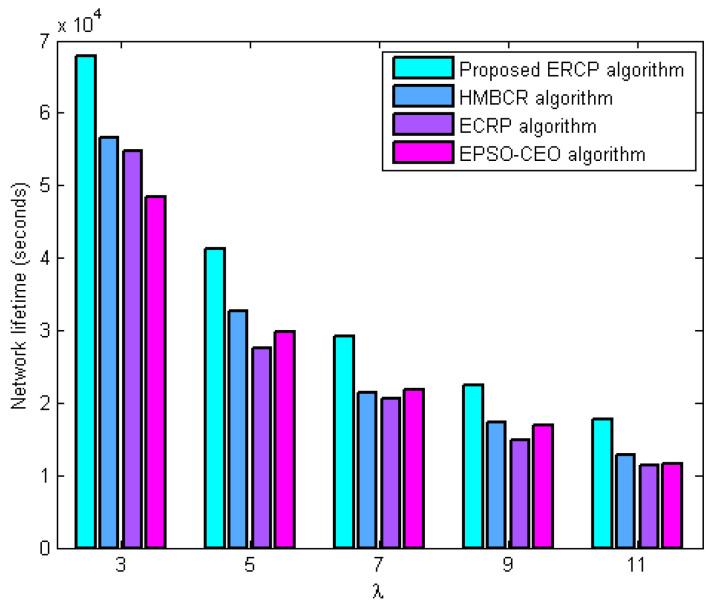
Influence of increasing average traffic rate on network lifetime for homogenous networks.

**Figure 2:**
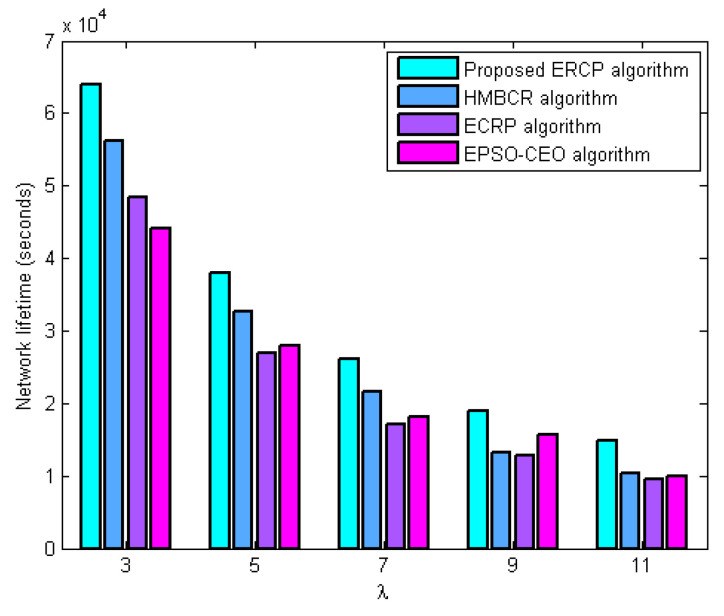
Influence of increasing average traffic rate on network lifetime for heterogeneous networks.

In this series of experiments, the performance of the proposed ERCP algorithm was evaluated in terms of the network’s lifetime in comparison to HMBCR [10], ECRP [11], and EPSO-CEO [24] for both homogeneous and heterogeneous networks under different traffic rates. This evaluation was carried out in terms of how long the network remained operational. Figure 1 and Figure 2 both illustrate the relationship between the average traffic rate and the variance in the lifetime of the network for homogeneous and heterogeneous networks, respectively. The figures demonstrate that the ERCP method, which was proposed, enhances the network’s lifetime in comparison to the HUMBCR algorithm, the ECRP algorithm, and the EPSO-CEO algorithm for networks that are either homogeneous or heterogeneous. Some of the reasons why such outcomes are justified are as follows:

The proposed ERCP algorithm balances network energy use among sensor nodes at the cluster head selection level due to the suggested energy measure. It also considers link quality when choosing a cluster head to cut down on energy wastage from retransmitting failed packets. HUMBCR, ECRP, and EPSO-CEO algorithms waste resources by retransmitting failed packets during cluster head selection because they are unaware of the reliability of data transfer from member nodes to cluster heads. That is to say, the obtained results further validate the benefits of the proposed quality metric. At the same time, it can achieve effective load balance among cluster heads through the proposed load metric. Therefore, the obtained results further validate the load metric’s benefits in network lifetime enhancement.

At the routing level, the proposed ERCP algorithm utilizes the node’s energy metric, which leverages node traffic load and residual energy to balance network energy usage across sensor nodes effectively. It prevents lost links to save energy wasted by retransmitting dropped packets as much as possible. On the other hand, HUMBCR, ECRP, and EPSO-CEO algorithms depend on residual energy to balance energy consumption, which is inadequate according to this work’s observations. They attempt to route data along the shortest path, but a lack of knowledge regarding data transmission reliability wastes energy by retransmitting missed packets.

#### 5.3.2. Packets Delivery Ratio (PDR) Evaluation

**Figure 3:**
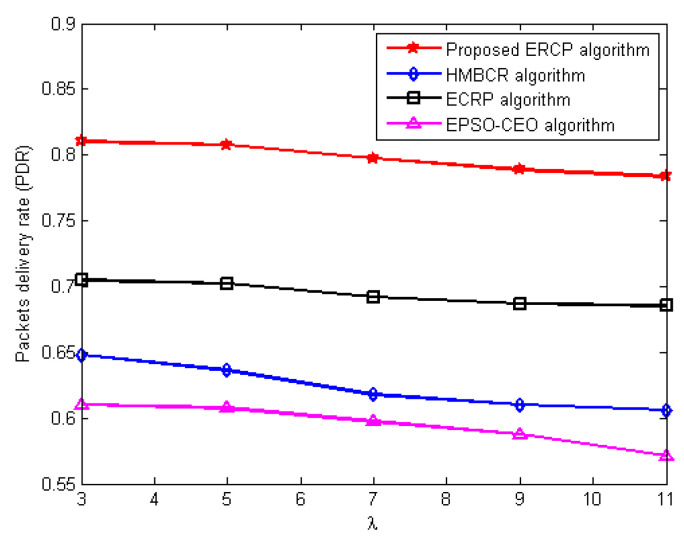
Influence of increasing average traffic rate on packets’ delivery ratio for homogenous networks.

**Figure 4:**
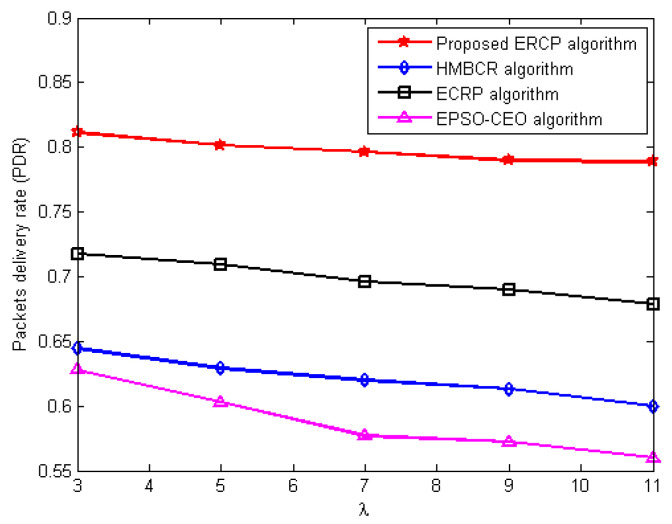
Influence of increasing average traffic rate on packets’ delivery ratio for heterogeneous networks.

This series of experiments is carried out with the intention of determining how well the ERCP proposed algorithm performs in comparison to the HMBCR [10], ECRP [11], and EPSO-CEO [24] algorithms in terms of the packet delivery ratio for homogeneous and heterogeneous networks operating at varying traffic rates. Figure 3 and Figure 4 demonstrate the variance in network PDR that occurs with the average traffic rate for networks that are either homogenous or heterogeneous, respectively. The figures show that the proposed ERCP algorithm produces the highest PDR compared to the HUMBCR, ECRP, and EPSO-CEO algorithms for both homogeneous and heterogeneous networks, even upon increasing the average traffic rate in the network. This enhancement is due to two main factors: The first is that the link quality is integrated into the selection decision of the cluster head, preventing the forwarding of data packets from member nodes to their cluster heads via unreliable links. That is to say, the obtained PDR is evidence that the proposed cluster head selection algorithm can improve network reliability through the proposed quality metric. The second factor is that the proposed routing algorithm reliably forwards the data packet by integrating the link quality into the routing decision.

In contrast, HUMBCR, ECRP, and EPSO-CEO algorithms did not take into account the reliable data transmission, whether from the member nodes to their cluster heads or from the cluster heads to the sink node, which increases the packet loss rate and thus diminishes the network throughput.

#### 5.3.3. Average End-to-End Delay Evaluation

**Figure 5:**
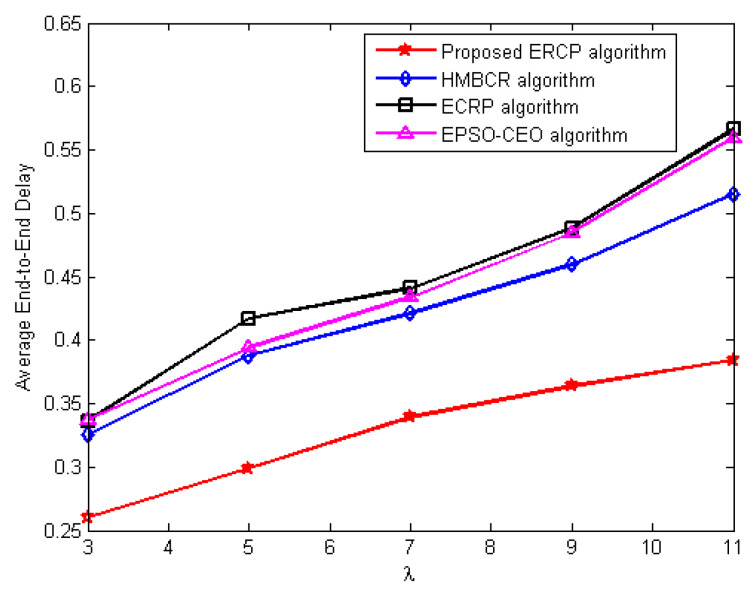
Influence of increasing average traffic rate on average end-to-end delay for homogenous networks.

**Figure 6:**
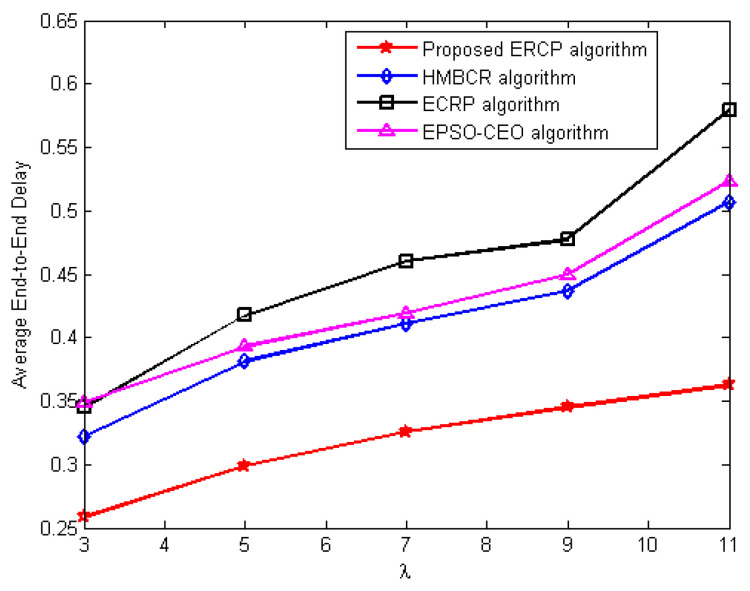
The influence of increasing average traffic rate on end-to-end delay for heterogeneous networks.

Another series of experiments are carried out to investigate the relationship between the typical end-to-end latency and the typical traffic rate. Comparisons are made between the HMBCR [10], ECRP [11], and EPSO-CEO [24] approaches for homogeneous and heterogeneous networks operating at varying traffic rates using the ERCP technique that was just proposed. The differences in the average end-to-end delay that occur with the average traffic rate are shown in Figure 5 and Figure 6 for homogeneous and heterogeneous traffic patterns, respectively.

The suggested ERCP has the lowest end-to-end latency compared to the HUMBCR, ECRP, and EPSO-CEO algorithms for both homogeneous and heterogeneous networks, as evident from the numbers below. It selects forwarding nodes that can send the packet through a more reliable connection, hence lowering the probability of packet loss and retransmission, which, in turn, lowers the delivery latency.

As mentioned above, in the case of the HUMBCR, ECRP, and EPSO-CEO algorithms, packets cannot bypass the lossy links, which results in an increase in the end-to-end latency due to the retransmission of lost packets.

#### 5.3.4. Energy Balance Evaluation

**Figure 7:**
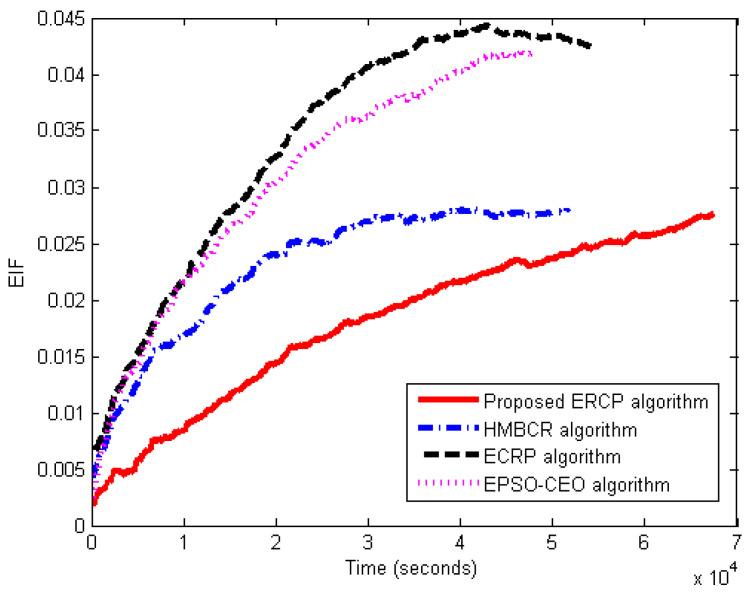
Energy Imbalance Factor (EIF) vs. the running time for homogenous networks.

**Figure 8:**
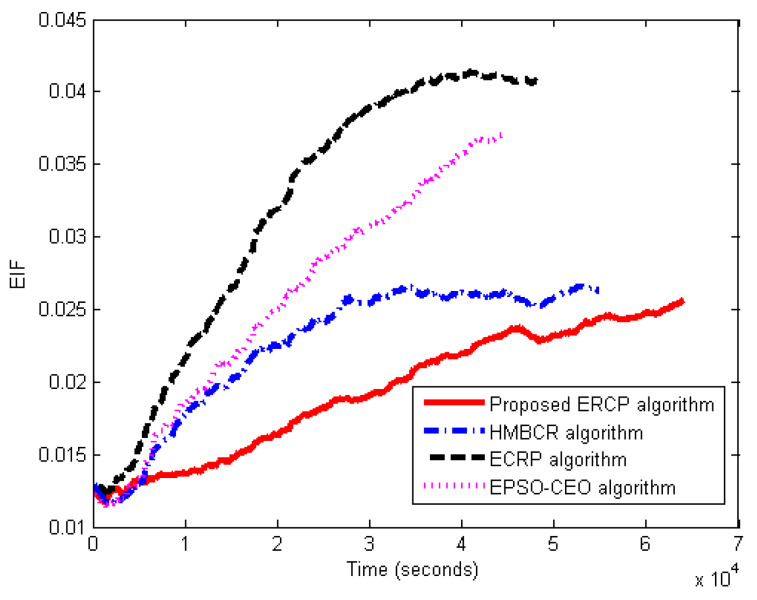
Energy Imbalance Factor (EIF) vs. the running time for heterogeneous networks.

This set of experiments is carried out for the proposed ERCP approach evaluation in terms of energy balance, EIF. The proposed ERCP approach is compared to HMBCR [10], ECRP [11], and EPSO-CEO [24] for both homogeneous and heterogeneous networks. The EIF was determined during the running time to obtain the network’s balance efficiency. The average traffic rate is set to three packets per second. Figure 7 and Figure 8 show the EIF variation with respect to the simulation time for networks of both a homogeneous and heterogeneous nature, respectively. From the figures, it is clear that with the increase in simulation time, the EIF increases. Indeed, the sink node neighbors are highly used more than the others. Undoubtedly, it is negatively affecting the energy variance across the network. It reveals the reason why EIF is augmented with more running time.

However, based on the results in Figure 7 and Figure 8, the proposed ERCP algorithm produced a lower EIF compared to the others. This means that the sensor nodes’ energy in the entire network using the proposed algorithm is closer to the average energy than that of the others. That is to say that the obtained EIF is evidence that the proposed ERCP algorithm can efficiently balance the energy usage compared to the other. In the case of HUMBCR, ECRP, and EPSO-CEO algorithms, the data packets have to be relayed via the nodes with the highest residual energy to balance energy consumption. According to the resulting EIF, it is evident that relying on residual energy is inadequate to achieve an appropriate energy balance. Therefore, this further validates the benefits of the proposed nodes’ energy metric to obtain a better energy balance level. This explains why the proposed ERCP algorithm balances network energy utilization more efficiently than the HUMBCR, ECRP, and EPSO-CEO algorithms.

#### 5.3.5. Complexity Evaluation

**Figure 9:**
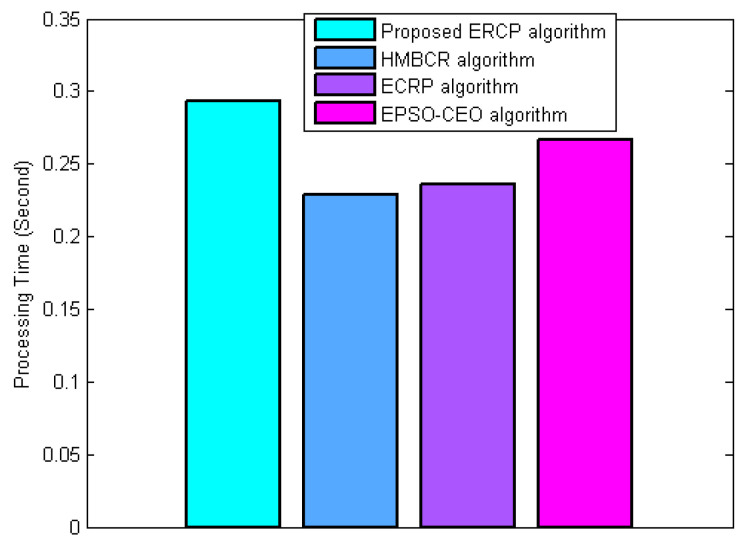
Overall complexity in terms of the required processing time.

Throughout this experiment, the overall complexity of the proposed algorithm is evaluated in terms of the processing time required in comparison to HMBCR [10], ECRP [11], and EPSO-CEO [24]. Figure 9 shows the overall complexity in terms of the processing time required. The figure shows that the proposed algorithm requires a longer processing time than the others. Therefore, the complexity of the proposed algorithm is higher than that of the other algorithms. That is to say, the proposed algorithm needs more computational energy. However, in WSNs, energy consumption in communication has been recognized as the main source of energy consumption, and it costs much more than the energy consumption of computation. However, this disadvantage in complexity is compensated for by the good performance in network PDR, network lifetime, delivery delay, and energy balance.

## 6. Conclusions

In this paper, an efficient ERCP protocol for maximizing the network’s lifetime and throughput has been developed. The ERCP protocol includes both the selection of the cluster head and the algorithms for inter-cluster data routing. The cluster head selection algorithm ensures the network’s most efficient selection of cluster heads. The clustering algorithm is based on four criteria: The distance to the sink node, distance to neighbors, quality metric, and residual energy. After clustering, an inter-cluster routing algorithm takes place for data transmission between the sensor nodes and the sink node. It finds the most efficient paths to the sink node so that it can receive the data. The decision regarding the routing is determined via a cost function that depends on three parameters: The node’s energy metric, the connection quality, and the distance to the sink node. Extensive simulation experiments were carried out to verify that the ERCP method is effective. The comparison results in terms of the network’s lifetime, PDR, average end-to-end latency, and EIF proved that the ERCP protocol outperformed earlier works such as HUMBCR, ECRP, and EPSO-CEO algorithms. The proposed protocol could be developed in the future considering the WSN with a mobile sink node.

## Figures and Tables

**Table 1:** A comparative summary of the above-mentioned algorithms and the proposed ones.

Name of the Algorithm	Advantages	Disadvantages
LEACH	Enhances energy efficiency by Periodically rotating the cluster heads.	Unbalanced energy consumption due to the random selection of the cluster heads, and it is not addressed the reliability issue.
FR-LEACH	Enhances energy efficiency by utilizing energy factor.	The load balancing issue is not fully addressed and the reliability issue is not considered.
DARE-SEP	Enhances energy efficiency by utilizing energy and distance factors.	The load balancing issue is not fully addressed and the reliability issue is not considered.
HMBCR	Enhances energy efficiency by utilizing energy, distance, and load factors.	The reliability issue is not considered.
ECRP	Enhances energy efficiency by utilizing energy and distance factors.	The load balancing issue is not fully addressed, and the reliability issue is not considered.
CRCGA	Enhances energy efficiency by utilizing energy, distance, and load factors.	The reliability issue is not considered.
CPMA	Enhances energy efficiency by utilizing energy and distance factors.	The load balancing issue is not fully addressed, and the reliability issue is not considered.
Greedy & GA ANN & GA	Enhances energy efficiency by utilizing energy and distance factors.	The load balancing issue is not fully addressed, and the reliability issue is not considered.
ML-TSEP	Enhances energy efficiency by utilizing energy, distance, and load factors.	The reliability issue is not considered.
DE-SEP	Enhances energy efficiency by utilizing energy, and distance factors.	The load balancing issue is not fully addressed, and the reliability issue is not considered.
GCEEC	Enhances energy efficiency and coverage.	The load balancing issue is not fully addressed, and the reliability issue is not considered.
EPSO-CEO	Enhances energy efficiency by utilizing energy and distance factors.	The load balancing issue is not fully addressed and the reliability issue is not considered.
The proposed ERCP	The energy efficiency, load balancing, and reliability issues are considered	Has more computation energy.

**Table 2:** Simulation environment parameters.

Parameters	Values
Deployment strategy	Uniformly random
Num sensor nodes	300
Maximum number of retransmissions	4
Packet size	50 byte
Buffer size	128 Kbyte
Frequency Path loss exponent	868 MHz 3
Minimum radio range	150 m
Data rate	20 Kbps
Shadow fading variance	3
Reference distance	1 m

## Data Availability

Not applicable.
